# Seed dispersal by monk sakis (*Pithecia monachus*)

**DOI:** 10.1007/s10329-026-01251-6

**Published:** 2026-04-02

**Authors:** Malika Gottstein, Katrin Heer, Eckhard W. Heymann

**Affiliations:** 1 Eva Mayr-Stihl Professorship for Forest Genetics, Faculty of Environment and Natural Resources, Albert-Ludwigs-Universität Freiburg, Freiburg im Breisgau, Germany; 2 Museum für Naturkunde – Leibniz Institute for Evolution and Biodiversity Science, Berlin, Germany; 3 Soziale Evolution der Primaten, Deutsches Primatenzentrum, Leibniz-Institut für Primatenforschung, Göttingen, Germany; 4 Verhaltensökologie, Johann-Friedrich-Blumenbach-Institut für Zoologie und Anthropologie, Fakultät für Biologie und Psychologie, Georg-August-Universität Göttingen, 37077 Göttingen, Germany

**Keywords:** Endozoochory, Frugivory, Seasonally flooded forest, Seed dispersal–predation continuum, Western Amazonia

## Abstract

Seed dispersal by animals traditionally considered seed predators challenges the classical dichotomy between antagonistic and mutualistic plant–animal interactions. Pitheciines are typically regarded as specialized seed predators due to their seed-masticating feeding behavior, yet evidence suggests they can also contribute to endozoochorous seed dispersal when seeds escape damage during ingestion. We investigated fecal samples of monk sakis (*Pithecia monachus*) in a seasonally flooded forest in western Amazonia over the course of one year, quantifying and identifying intact seeds recovered from the samples. Of 92 fecal samples analyzed, 54% contained one or more intact seeds, with up to 17 seeds and three morphospecies per sample. In total, we recovered 165 intact seeds representing 20 morphospecies, all small-seeded taxa with a maximum seed length of < 1 cm. Fresh fecal pellets float on the water surface in flooded forest habitats, suggesting a potential interaction between endozoochorous and hydrochorous dispersal. Although we did not assess seed viability or germination, our results demonstrate that monk sakis can contribute to seed dispersal through gut passage and place them along the seed dispersal–predation continuum. These findings highlight the need to further investigate the dispersal potential and ecological significance of seed-predating primates.

## Introduction

Seed dispersal is a fundamental ecological process shaping plant spatial distribution, population dynamics, and long-term persistence (Nathan and Muller-Landau 2000; Wang and Smith 2002). Since successful dispersal can improve plants’ reproductive success by reducing density-dependent mortality and enhancing seedling establishment (Janzen 1970; Connell 1971), many plants invest in attracting animals to transport their seeds (Jordano 2000). Traditionally, frugivores have been classified as either seed dispersers, which swallow seeds whole and deposit them intact, or seed predators, which destroy seeds through mastication (van Leeuwen et al. 2022). The consequences for plants appear fundamentally different under this dichotomy: dispersers provide clear benefits to plants, whereas predators impose only costs (Janzen 1971; Norconk et al. 1998). However, this dichotomous framework oversimplifies the complexity of plant–animal interactions (Norconk et al. 1998; Heleno et al. 2011). An increasing number of studies shows that fruit-eating animals actually fall along a seed dispersal–predation continuum, rather than forming two discrete categories (Dracxler and Forget 2017; Montesinos-Navarro et al. 2017; Gómez et al. 2019; van Leeuwen et al. 2022; Chen et al. 2023). Many species typically regarded as dispersers also destroy some proportion of the seeds they ingest (e.g. tapirs (Bodmer 1991), bats (Wagner et al. 2015)), while species considered predators may contribute to dispersal when seeds escape damage and are defecated or dropped (e.g. pitheciine and colobine primates (Norconk et al. 1998; Tsuji et al. 2017)), birds (Heleno et al. 2011). Moreover, the balance between seed destruction and dispersal can vary temporally and spatially, depending on the environmental context (Perea et al. 2013; González-Varo et al. 2019).

Primates, which are predominantly frugivorous, play a central role in tropical seed dispersal and strongly influence forest regeneration dynamics (Fleming and Kress 2013; Hawes et al. 2024). At the same time, many primates exploit seeds as a food resource, placing them along the seed dispersal–predation continuum rather than exclusively in one category (Norconk et al. 2013). Pitheciines are usually considered typical seed predators because they masticate seeds (Kay et al. 2013). Their morphological and behavioral adaptations allow them to access and digest seeds, thereby destroying them and suggesting a predominantly antagonistic role (Kinzey and Norconk 1990; Norconk and Veres 2011; Ledogar et al. 2013). However, pitheciines may also play a role in seed dispersal. They can disperse seeds by manipulating and dropping them during feeding, and in some cases, small seeds may escape mastication and are swallowed intact, and thus can be dispersed through endozoochory (Norconk 2021). Evidence for this is limited but notable: Norconk et al. (1998) reported intact seeds of four plant species (all < 2 mm in length) in the feces of white-faced sakis (*Pithecia pithecia*) and black bearded sakis (*Chiropotes satanas*); Port-Carvalho and Ferrari (2004) found that the latter species dispersed seeds from nine plant families, with seed lengths ranging from 1 to 20 mm; and van Roosmalen et al. (1988) also found intact seeds of four plant species in feces of reddish-brown bearded sakis (*Chiropotes sagulatus*, previously *Chiropotes satanas chiropotes*).

To further explore the role of pitheciines in seed dispersal, we collected fecal samples of monk sakis (*Pithecia monachus*) in a seasonally flooded forest in western Amazonia, where their feeding ecology has been described previously (Gottstein et al. 2023). We examined fecal deposition characteristics, as well as the quantity and identity of seeds present. Our aim was to determine whether monk sakis disperse seeds through gut passage.

## Methods

The study was conducted in the Área de Conservación Regional Comunal Tamshiyacu-Tahuayo (ACRCTT) in northern Peruvian Amazonia in the department of Loreto. We collected data around the Amazon Research Center (ARC; 4°22′23′′–4°24′16′′S, 73°14′45′′–73°16′36′′W), located in the floodplain of the Tahuayo River, a tributary of the Amazon River. The study site is seasonally flooded, with flooding beginning at the end of January and peaking from late March through May (Gobierno Regional de Loreto 2009; Ronchail et al. 2018). Fieldwork was conducted continuously from July 2019 to July 2020. We followed 12 groups of monk sakis (*Pithecia monachus*) within the ACRCTT and its buffer zone. Group size ranged from two to seven individuals, with most groups consisting of five individuals (median = mean = 5). Each group contained at least one adult female and one adult male, and juveniles were present in 11 groups during the study period. The monk sakis were not habituated to human observers and were generally very shy and difficult to observe. As a result, continuous observations were often challenging, and contact durations with groups were generally short. Once we located a group, we followed it as long as possible, recording the dietary composition (Gottstein et al. 2023), and collecting fecal samples opportunistically immediately after defecation.

We placed fecal samples into plastic containers, flattened to facilitate rapid drying and ethanol penetration, and submerged in 96% ethanol for at least 48 h. Each sample corresponded to a single defecation event by one individual and consisted of one or several pellets. When multiple pellets were produced during a single defecation event, they were pooled and treated as one sample. After ethanol preservation, we stored the samples dry over silica gel. In the laboratory, we softened samples in water for approximately one hour, then extracted seeds using forceps. We counted only seeds that appeared intact, defined as retaining their original shape and seed coat and showing no visible cracks or fractures. For each intact seed, length and width were measured using a standard linear ruler. We first classified the extracted seeds into morphospecies and then identified them following Cornejo and Janovec (2010), using their identification key, family- and genus-level descriptions, and photographic plates. Seed morphology was compared systematically to descriptions and images in the reference to assign taxonomic identities whenever possible.

## Results

**Fig. 1:**
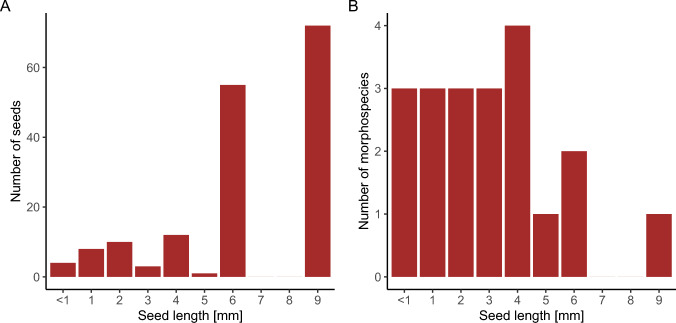
Seed size distributions in the feces of monk sakis. **A** Size distribution of all intact seeds (n = 165) recovered from fecal samples. Bars show the number of seeds in each length category. **B** Size distribution of intact plant morphospecies (n = 20) detected in fecal samples. Bars show the number of morphospecies occurring in each length category

We examined 92 fecal samples. Each sample contained up to five small, hard pellets (~ 0.5 cm × 1 cm), and we observed no seeds on the surface of the pellet during field collection. During field collection, we observed that freshly defecated monk saki feces floated on the water surface in flooded forest habitats. Fifty samples (54%) contained one or more intact seeds. Among the seed-containing samples, the number of seeds ranged from 1 to 17, representing up to three morphospecies per sample. All recovered seeds were small, with a maximum length of up to 9 mm (Fig. 1). In total, we counted 165 intact seeds across all fecal samples. We identified a total of 20 seed morphospecies, and 10 of the them to genus or family level (Table 1).

**Table 1:** Seeds recovered from monk saki (*Pithecia monachus*) fecal samples (n = 92)

Taxon	Number of dispersed seeds	(Min. -) max. seed length [mm]	(Min. -) max. seed width [mm]
Annonaceae	72	(6 -) 9	(2 -) 3
Boraginaceae	47	(4 -) 6	(3 -) 5
*Philodendron* (Araceae)	5	(4 -) 4	(1 -) 1
*Cissus* (Vitaceae)	3	(3 -) 4	(3 -) 4
*Norantea* (Marcgraviaceae)	3	(4 -) 4	(1 -) 1
*Anthurium* (Araceae)	1	3	1
*Cecropia* (Urticaceae)	1	4	1
*Passiflora* (Passifloraceae)	1	6	4
*Vismia* (Clusiaceae)	1	5	1

## Discussion

Our results demonstrate that monk sakis, typically regarded as seed predators, can also contribute to seed dispersal through gut passage. We recovered intact seeds from approximately half of the of fecal samples, indicating that seed survival during ingestion may be more common than previously assumed and endozoochorous dispersal by sakis can be beneficial for plants.

The taxa of seeds recovered from monk saki feces partially overlapped with the plant species we identified in our feeding ecology study based on behavioral observations at the same field site (Gottstein et al. 2023). However, fecal analyses also revealed additional plant families that direct feeding records had not captured. For example, we recovered seeds from Annonaceae, a family reported as an important food resource for sakis in other studies but not recorded in our observational dataset (Peres 1993; Setz 1993; Gottstein et al. 2023). This suggests that fecal analyses may reveal interactions that are easily overlooked in observational studies, particularly when seeds are small, ingested in small quantities and from epiphytes.

We found that the proportion of fecal samples containing intact seeds was surprisingly high. Studies of typical seed dispersers often report seeds in nearly all fecal samples (e.g., Link and Di Fiore (2006) on spider monkeys, Heymann et al. (2022) on tamarins). However, even among neotropical primates generally considered seed dispersers, intact seeds are not present in all fecal samples, as shown for spider monkeys, howler monkeys and woolly monkeys (Chapman 1989; Stevenson 2000; Chaves et al. 2012). In this context, the proportion of monk saki fecal samples containing seeds, while lower than that of a typical disperser, is not negligible. A key difference may lie in the size spectrum of dispersed seeds: monk sakis appear to disperse primarily small seeds, with an upper size limit constrained by their seed-masticating feeding behavior. This pattern is consistent with previous reports that even dedicated seed predators can allow small seeds to escape mastication and pass intact through the gut (van Roosmalen et al. 1988; Norconk et al. 1998).

In seasonally flooded forests, seed dispersal by monk sakis may have important ecological consequences due to the potential for hydrozoochory. We observed that monk saki feces float on water, creating the potential for seeds contained within fecal pellets to combine endozoochorous dispersal with hydrochorous transport. Hydrochory is known to facilitate long-distance seed movement in floodplain systems, potentially enhancing gene flow and colonization of changing habitats (Wittmann and Householder 2017). In this context, even low rates of seed survival and dispersal by sakis could contribute meaningfully to plant spatial dynamics. Of all other primates inhabiting seasonally flooded forests at our site (see Hores (2018) for a list or primate species at the ARC), the following are known or supposed seed dispersers: *Leontocebus nigrifrons, Saguinus mystax, Plecturocebus cupreus, Cebus unicolor, Sapajus macrocephalus, Saimiri macrodon* and *Aotus nancymaae* (Peres and van Roosmalen 2002; Helenbrook et al. 2020; Heymann et al. 2022); they may similarly interact with flood-mediated transport processes. However, whether feces of these species float and facilitate subsequent hydrochorous transport has not yet been evaluated.

Our results support the idea that animals often considered seed predators can still contribute to seed dispersal. By examining monk sakis as part of the wider spectrum of plant–frugivore interactions, we gain a clearer picture of their ecological role and the variety of dispersal pathways in Neotropical forests. However, we did not assess seed viability or germination, and therefore cannot determine whether intact seeds remained capable of successful establishment. In addition, we did not evaluate post-dispersal processes such as secondary dispersal and predation after defecation (e.g., by fish (Horn et al. 2011)), leaving the ultimate fate of dispersed seeds unresolved. Consequently, we cannot assess the overall effectiveness of monk sakis as seed dispersers, nor determine whether gut-passed seeds ultimately contribute to plant recruitment (Schupp 1993; Schupp et al. 2010). Future studies combining fecal analyses, feeding observations, and seed fate experiments will be necessary to quantify dispersal distances, post-dispersal survival, and recruitment success of seeds dispersed by monk sakis.
